# Validation of SUV thresholds in [¹⁸F]SiTATE PET/CT for accurate meningioma segmentation

**DOI:** 10.1007/s00259-025-07476-9

**Published:** 2025-07-23

**Authors:** Nabeel Mansour, Julian Joram, Freba Grawe, Anna Hinterberger, Johannes Rübenthaler, Konstantin Klambauer, Wolfgang G. Kunz, Michael Winkelmann, Clemens C. Cyran, Jens Ricke, Osman Öcal, Marcus Unterrainer, Klaus Jurkschat, Carmen Wängler, Björn Wängler, Ralf Schirrmacher, Alexander Nitschmann, Tobias Greve, Gabriel Sheikh, Adrien Holzgreve, Nathalie L. Albert, Matthias P. Fabritius

**Affiliations:** 1 Department of Radiology, LMU University Hospital, LMU Munich, Marchioninistr. 15, 81377 Munich, Germany; 2 DKFZ Hector Cancer Institute at the University Medical Center Mannheim, 69120 Heidelberg, Germany; 3 Department of Clinical Radiology and Nuclear Medicine, Medical Faculty Mannheim, University Medical Center Mannheim, Heidelberg University, 68167 Mannheim, Germany; 4 Department of Diagnostic & Interventional Radiology, Heidelberg University Hospital, Heidelberg, Germany; 5 Department of Nuclear Medicine, LMU University Hospital, LMU Munich, Munich, Germany; 6 DIE RADIOLOGIE, Munich, Germany; 7 Fakultät für Chemie und Chemische Biologie, Technische Universität Dortmund, Dortmund, Germany; 8 Biomedical Chemistry, Department of Clinical Radiology and Nuclear Medicine, Medical Faculty Mannheim of Heidelberg University, Mannheim, Germany; 9 Molecular Imaging and Radiochemistry, Department of Clinical Radiology and Nuclear Medicine, Medical Faculty Mannheim of Heidelberg University, Mannheim, Germany; 10 Department of Oncology, Division of Oncological Imaging, University of Alberta, Edmonton, AB Canada; 11 Department of Radiation Oncology, LMU University Hospital, LMU Munich, Munich, Germany; 12 Department of Neurosurgery, LMU University Hospital, LMU Munich, Munich, Germany

**Keywords:** PET/CT, Meningioma, SiTATE, Threshold, Segmentation

## Abstract

**Purpose:**

Somatostatin receptor (SSTR)-targeted PET/CT provides valuable clinical insights beyond standard imaging in meningioma patients. Due to its excellent diagnostic capabilities and favorable logistics, the ^18^F-labeled SSTR-targeting peptide SiTATE is increasingly in demand. We aimed to validate a recently proposed standard uptake value (SUV) threshold for accurate meningioma delineation in a clinically diverse patient cohort, including complex anatomical locations and lesions with prior surgical intervention.

**Methods:**

Consecutive patients with known or suspected meningioma who underwent [^18^F]SiTATE PET/CT and contrast enhanced cerebral MRI were included. Lesions were semi-automatically segmented on PET images using an individualized minimal SUV (SUV_min_) within a manually defined volume of interest. Correlative CT and MRI images were used to refine segmentations for each lesion, identifying the optimal lesion-specific SUV_min_ to accurately capture the true volume of the meningioma. All lesions were additionally segmented using the recently proposed threshold of 4.0, and resulting volumes were compared.

**Results:**

61 patients with 109 lesions were analyzed: 40 (37%) extraosseous, 32 (29%) partial trans-osseous, and 37 (34%) predominantly intraosseous. The median optimal SUV_min_ for lesion delineation was 4.2. Osseous involvement did not significantly affect the median SUV_min_ (*p* = 0.1). Individualized SUV volumes showed excellent absolute agreement with those obtained using the fixed threshold of 4.0 (ICC[A,1] = 0.967; 95% CI: 0.952–0.977; *p* < 0.0001). However, 17 lesions (SUV_max_ < 4.2) were not captured by the fixed threshold.

**Conclusion:**

The proposed SUV threshold of 4.0 showed promising results, supporting its suitability for clinical practice. Although limitations were evident, with 16% of lesions — primarily very small — showing reduced uptake and therefore not captured by this threshold, the study underscores its applicability in clinical practice.

## Introduction

Meningiomas are the most commonly reported primary intracranial, extra-cerebral neoplasms [1, 2]. Despite limited controlled clinical trials, recent advances are refining meningioma management regarding diagnostic and therapeutic approaches. The updated European Association of Neuro-Oncology (EANO) guidelines emphasize MRI for provisional diagnosis, often managed by watch-and-scan for asymptomatic or elderly patients. Definitive diagnosis requires surgical intervention, which is often curative, while patients with inoperable or partially resected tumors, higher tumor grades or recurrent tumors may benefit from radiosurgery, fractionated radiotherapy (RT) or medical therapy [3].

Somatostatin receptor (SSTR)-directed positron emission tomography (PET) provides high sensitivity and specificity for meningioma detection, offering insights beyond MRI or CT [4, 5]. It aids in differential diagnosis, tumor delineation (i.a. for radiotherapy planning), detection of osseous involvement, and in distinguishing scar tissue from recurrence [6–8]. Additionally, SSTR-based peptide receptor radionuclide therapy (PRRT) is emerging as a potential treatment [9, 10]. Regarding tumor segmentation, PET/CT contributes to accurate lesion delineation by providing objective, functional information that complements anatomical imaging. This is particularly valuable for radiotherapy planning and in anatomically complex regions, such as the skull base, or in postoperative and post-radiotherapy settings, where CT and MRI alone may be insufficient to reliably differentiate viable tumor tissue from scar or treatment-related changes.

The standard SSTR-PET ligands, [^68^Ga]Ga-DOTATATE and [^68^Ga]Ga-DOTATOC, are commonly used for neuroendocrine tumor and meningioma imaging but are limited by costly ^68^Ge/^68^Ga-generators, small batch production, short half-life, and lower image resolution compared to other isotopes, especially [^18^F] [11]. [^18^F]SiTATE, a new SSTR-targeting peptide, addresses these issues and has shown promising preliminary results in imaging of neuroendocrine tumors and meningiomas [12–17].

The recently published joint practice guideline, which was collaboratively developed by EANO, the European Association of Nuclear Medicine (EANM), the Society of Nuclear Medicine and Molecular Imaging (SNMMI), and the PET task force of the Response Assessment in Neurooncology Working Group (PET/RANO), established standards for PET imaging and SSTR-targeted PRRT in meningioma care, aiming to harmonize procedures, improve data comparability across centers, and support the collection of larger databases [18]. Nevertheless, standardized uptake value (SUV) thresholds in SSTR-PET imaging for meningiomas– particularly valuable for (semi-)automated tumor segmentation– have yet to be established, limiting their broader application in diagnosis, therapy planning, and treatment monitoring. While some studies have suggested SUV thresholds for [^68^Ga]Ga-DOTATATE PET, no standardized values have been proposed for [^18^F]SiTATE until recently [8, 19]. Kunte et al. demonstrated that a fixed SUV threshold of 4.0 strongly correlates with the CT-derived volumetric reference standard for delineating extraosseous meningiomas in a very small and selective patient cohort with clearly defined meningiomas and no suspicion of intraosseous expansion [20].

This study aims to build on these findings by validating the proposed threshold in a larger, clinically diverse cohort. In contrast to previous work, our analysis includes meningiomas with osseous involvement, as well as lesions in complex anatomical locations and postoperative sites, all scenarios that better reflect real-world clinical conditions and where accurate segmentation remains particularly challenging.

## Methods

### Patients

We retrospectively analyzed all patients with known or suspected meningioma who underwent [^18^F]SiTATE PET/CT and a matching contrast enhanced cerebral MRI (CE-MRI) at our center between 2020 and 2023, including those who did not receive treatment, those with suspected recurrence or tumor remnants and patients scheduled for radiotherapy planning. Patients were referred by neurosurgeons or radiation oncologists. Lesions previously treated with radiotherapy were excluded due to a potential impact on SSTR-expression. All patients gave written consent to undergo [^18^F]SiTATE PET/CT according to the regulations of the German Pharmaceuticals Act § 13(2b). This study complied with the Declaration of Helsinki and its amendments and was approved by the institutional ethics board of Ludwig Maximilians University (LMU) Munich (IRB 22–0353).

### PET/CT imaging parameters

SiTATE was obtained from ABX, Advanced Biomedical Compounds (Dresden, Germany). Radiosynthesis of [^18^F]SiTATE was performed at the Department of Nuclear Medicine, LMU Munich, Germany, as described in previous studies [12, 21–23]. All quality control measurements met the local product release criteria. After intravenous injection of [^18^F]SiTATE, PET scans were acquired with a median dose of 147 Megabecquerels (MBq) (IQR: 44 MBq) at 90 min after injection, with a scan duration of 15–20 min. If no medical contraindications were present, patients were additionally premedicated with furosemide (20 mg/2 mL injection solution; ratiopharm GmbH, Ulm, Germany) for radiation protection [24]. PET/CT included diagnostic, contrast-enhanced CT (CE-CT) scans with 1.5 mL of iopromide (Ultravist-300, Bayer Healthcare, Leverkusen, Germany) per kilogram of body weight. With CT scans serving for morphological correlation and attenuation correction, PET images were reconstructed with a transaxial 200 × 200 matrix using TrueX (including TOF, 2 iterations, and 21 subsets, and a 3D Gauss18 post-filter of 4-mm full width and at half maximum). All [^18^F]SiTATE PET/CT scans were acquired at the Department of Nuclear Medicine, LMU Munich, on a Siemens Biograph mCT flow or Siemens Biograph 64 (Siemens Healthineers, Erlangen, Germany) with a spatial resolution of 5.4 × 5.4 × 6.0 mm. The PET/CT scanner and reconstruction protocols used in this study fulfill the EANM/EARL2 standards.

#### Image analysis and segmentation

Image analysis was performed using a dedicated software package (Hermes Hybrid Viewer, Affinity 1.1.4; Hermes Medical Solutions, Stockholm, Sweden). First, all meningioma-typical lesions were identified on PET/CT by consensus of two board-certified radiologists with extensive experience in hybrid and neuro-oncologic imaging. To minimize inter-observer variability, all manual refinements of the SUVmin threshold were performed in consensus by the two radiologists. Both readers jointly reviewed each lesion in correlation with CE-CT and CE-MRI data to ensure consistent interpretation. This approach was intended to ensure reproducibility and reduce subjectivity in the manual refinement of lesion-specific SUVmin thresholds.

**Fig. 1:**
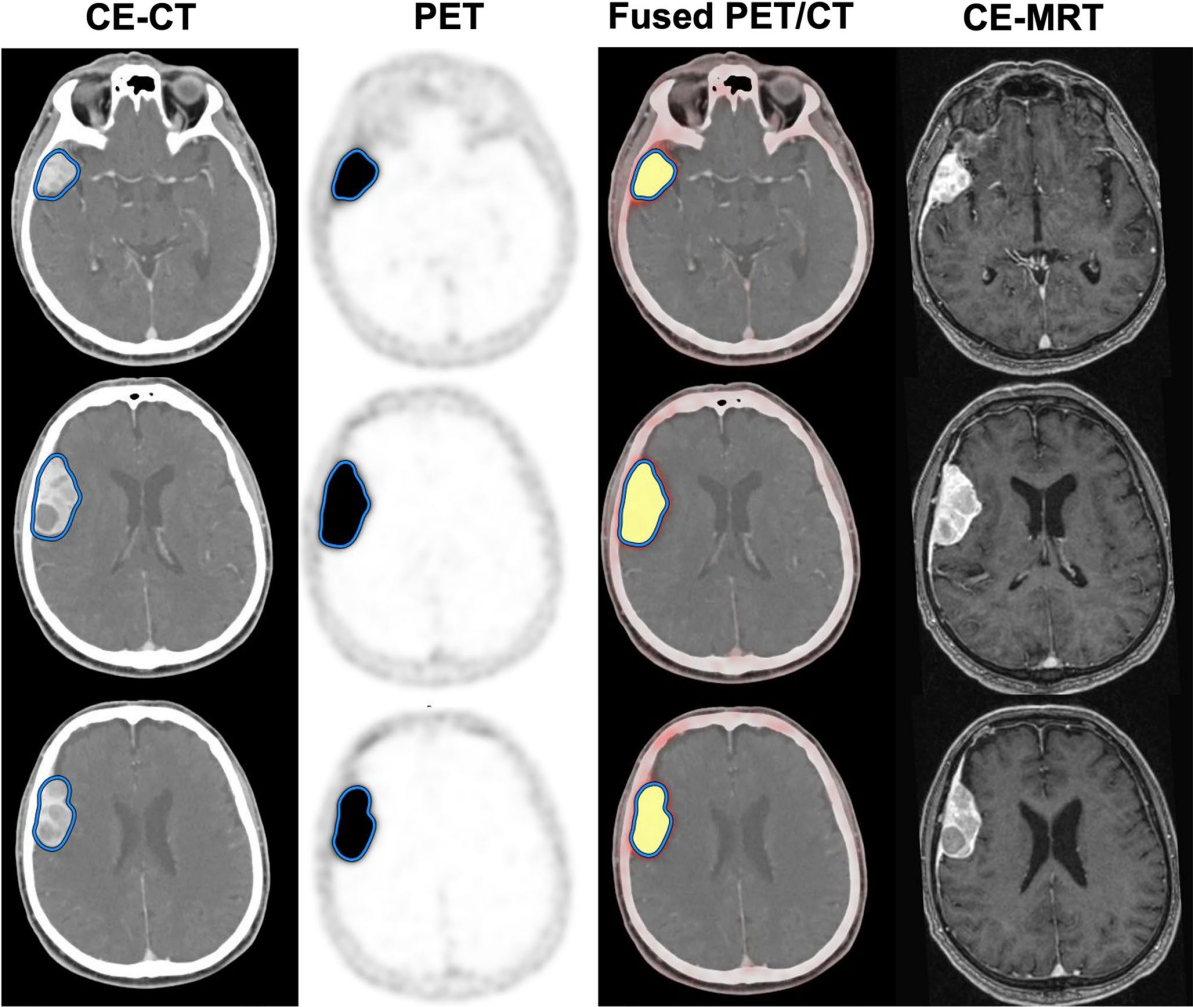
Example of SUV-thresholding for an extraosseous meningioma located in the right temporal region, prior to any treatment. The images are displayed in four columns, showing different modalities at the same axial level. From left to right: CE-CT, PET scan, fused PET/CT image, and corresponding CE-MRI on the far right

**Fig. 2:**
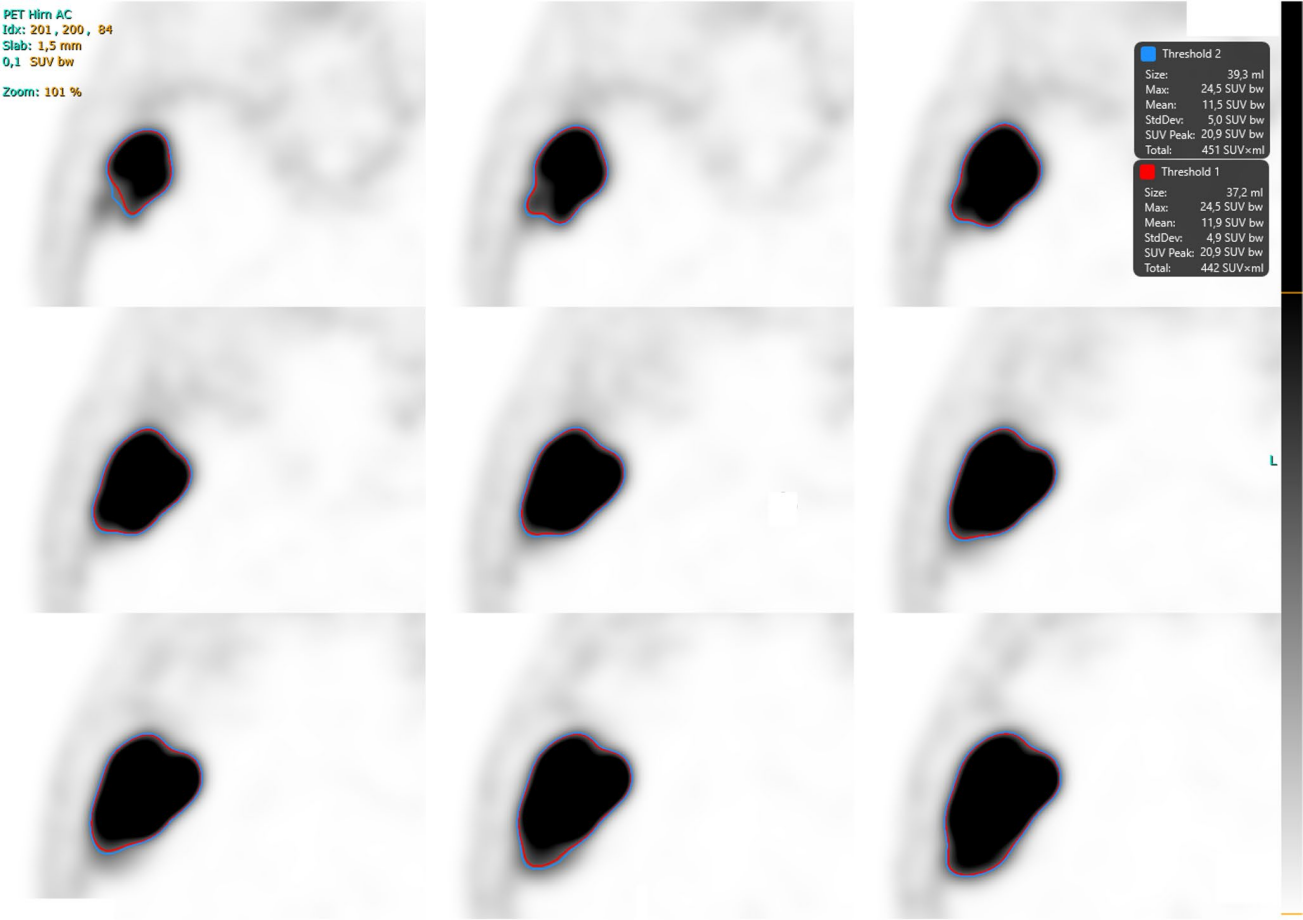
PET images showing 3D segmentations of the same extraosseous meningioma presented in Fig. 1, using an individualized threshold of 4.5 (red contour) and a fixed threshold of 4.0 (blue contour). The substantial overlap between segmentations demonstrates a high degree of concordance between the two methods, with only minimal differences in segmented volumes (39.3 ml vs. 37.2 ml)

Each lesion was initially segmented semi-automatically by placing a large, coarse volume of interest (VOI) on the attenuation-corrected (AC) PET component. For each lesion, the minimum standardized uptake value (SUV_min_) threshold was then manually adjusted upward or downward within the VOI to refine the segmentation mask. This refinement aimed to ensure the segmented region closely matched the true shape, volume, and extent of the lesion. The “true” lesion volume was established using an integrated ground truth derived from a comprehensive multimodal imaging assessment, incorporating CE-CT, CE-MRI, and PET component (Fig. 1), thereby establishing a lesion-specific optimal SUV_min_ threshold. A visual comparison between this individualized approach and a fixed threshold segmentation is illustrated in Fig. 2, highlighting the concordance between methods. Background activity in healthy/unaffected organs (pituitary gland, subarachnoid space and unaffected contralateral osseous regions) was also assessed using either a spherical VOI (subarachnoid space and unaffected contralateral osseous regions) or SUV-thresholding (delineation of the pituitary gland) in the same method used for meningiomas. The mean (SUV_mean_) and maximal standardized uptake values (SUV_max_) for all segmented structures were analyzed. In the case of meningiomas of the skull base in anatomic proximity to the pituitary gland, the respective VOI was refined manually in careful correlation with morphological imaging. The segmentation of the osseous component of the involved structures was achieved through Hounsfield unit (HU) thresholding, applied to the predefined volume guided by SUV_min_, with a specific HU range of 300–3000, adjusted to include highly dense cortical bone. As SUV_min_ was applied to segment the entire lesion on PET, no separate SUV_min_ values were calculated for the osseous or extraosseous compartments. These were subsequently delineated within the full lesion volume after CT-based HU thresholding. In cases where dense, non-osseous structures, such as pronounced parafalcine calcifications, were present, these were meticulously excluded to ensure accurate delineation of the osseous elements.

The contralateral subarachnoid space served as background for extraosseous parts of the meningioma. Meningiomas were categorized based on osseous involvement after a careful review of functional and morphological imaging as follows: (I) no osseous extension, (II) partial trans-osseous extension, and (III) predominant intraosseous extension, in accordance with previous literature [6, 12]. In all cases, PET information was directly correlated to CE-CT and CE-MRI. When obvious exceedance of dural and bone structures as defined by morphological imaging was present on PET, a partial trans-osseous involvement was noted. If PET imaging showed uptake restricted to the meningioma without any suspicion of extension to osseous structures confirmed by the absence of segmentation when applying the HU threshold, the lesion was classified as an extraosseous meningioma. In the case of SSTR expression with predominant intraosseous extension, the lesion was rated as predominantly intraosseous meningioma. In each patient, PET/CT findings suggestive of meningioma were compared with previous medical records and imaging to determine if the lesions were known or previously missed. The final classification of meningioma or non-meningioma was based on a consensus review of PET/CT findings, medical history, histological data (if available), and follow-up imaging when histology was not obtained.

### Statistical analysis

Data analysis was conducted using R software (version 4.4.1). Descriptive statistics were calculated and are presented as either median values with interquartile ranges (IQR) or mean values accompanied by standard deviation (SD). Group differences were assessed using Mann-Whitney U (for two groups) and Kruskal-Wallis tests (for multiple groups), which compare distributions based on ranked data. For SUV metrics related to osseous components (e.g., Osseous SUVmax and SUVmean), Kruskal–Wallis tests were performed only after excluding lesions without osseous involvement, to ensure that only biologically meaningful values were included in the analysis. In this study, it was applied to assess differences in SUV values and volumes of meningiomas across various intracranial locations. The intraclass correlation coefficient (ICC) was used to evaluate the consistency or agreement between measurements. A two-tailed p-value of less than 0.05 was considered statistically significant, indicating that any observed differences are unlikely to have occurred by chance.

## Results

### Patients and meningioma characteristics

A total of 109 lesions in 61 patients were included in the study. Of these, 45 patients were female (74%) and 16 were male (26%), with a mean age of 55.4 ± 3.7 years (Table 1). All patients underwent [^18^F]SiTATE PET/CT and CE-MRI, with a mean interval of 52 ± 24 days between the two scans. A histologically confirmed diagnosis was available for 60 of 61 patients (98%). Among these cases, 39/60 (65%) were classified as WHO grade 1 based in the World Health Organization (WHO) classification of the central nervous system (CNS), 19/60 (32%) as CNS WHO grade 2, and 2/60 (3%) as CNS WHO grade 3. None of the patients had histologically confirmed non-meningioma lesions. One patient exhibited characteristic imaging findings of meningioma in PET/CT and CE-MRI (intense homogeneous contrast enhancement and SSTR expression, dural tail sign) but opted for radiation therapy as the first line of treatment, and therefore no histological confirmation was obtained. Status post incomplete resection with residual meningioma tissue was present in 45 (75%) cases, while recurrent meningioma was observed in 15 (25%) cases. In addition to these treated meningiomas, subclinical or incidental untreated meningiomas were identified in 21 out of 61 patients (34%), with a total of 48 out of 109 lesions (44%) analyzed in this study. One patient presented with disseminated meningiomatosis. Of the meningiomas analyzed, 40 (37%) lesions were classified as extraosseous, 32 (29%) as partial trans-osseous, and 37 (34%) as predominantly intraosseous.

**Table 1:** Patient and lesion characteristics

Parameters	
Patients (*n* = 61)	
Age	55.4 ± 3.7 years
Sex	
Female	45 (74%)
Male	16 (26%)
Histology available	
CNS WHO grade 1	39 (65%)
CNS WHO grade 2	19 (32)
CNS WHO grade 3	2 (3%)
Lesions (*n* = 109)	
Location	
Convexity	37 (34%)
Sphenoid wing	31 (28%)
Parafalcine/Parasagittal	25 (23%)
Cerebellopontine	5 (4.5%)
Suprasellar/Parasellar	5 (4.5%)
Skull (intraosseous)	6 (6%)
Pretreatment prior to PET/CT	
None	50 (46%)
Surgery	59 (54%)
Radiotherapy	0
State of meningioma at timepoint of imaging	
Primary	50 (46%)
Residual	44 (40%)
Recurrent	15 (14%)
Extension	
extraosseous	40 (37%)
Partial trans-osseous	32 (29%)
Predominant intraosseous	37 (34%)

*CNS*, central nervous system, *WHO* World Health Organization

### Lesion characteristics

**Fig. 3:**
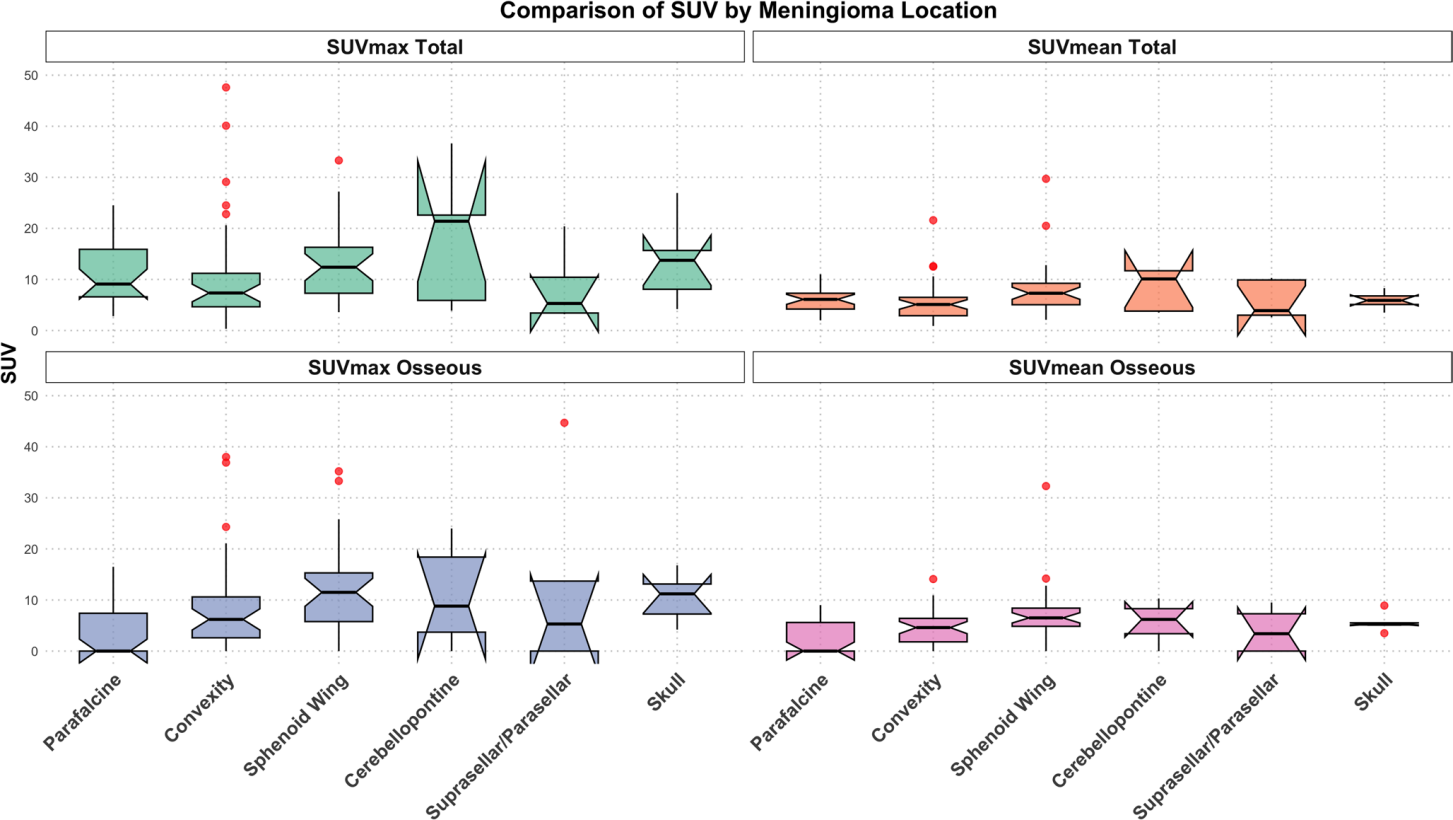
Distribution of Standardized Uptake Values (SUV) across different anatomical locations of meningiomas, including the median SUV_max_ and SUV_mean_ for both total and intraosseous parts of meningiomas. Osseous segmentation was performed using HU-thresholding (300–3000 HU) within the segmented volume defined by the individualized SUV_min_. Each boxplot illustrates the variability and central tendency of SUV values at distinct anatomical sites, highlighting differences in uptake based on the meningioma location. The notches in the boxplots represent the confidence interval around the median, providing a visual indication of the variability in the data. A non-overlapping notch between two boxplots suggests a statistically significant difference in the medians of the two groups

Overall, the median meningioma volume was 1.52 cm³ (IQR: 7.0 cm³), with a median SUV_max_ of 9.3 (IQR: 10.4) and a median SUV_mean_ of 5.9 (IQR: 4.3). The overall median optimal threshold (SUV_min_) for lesion delineation was 4.2 (IQR: 1.9). In extraosseous meningiomas, the median volume was 0.73 cm³ (IQR: 2.4 cm³), with a median SUV_max_ of 7.7 (IQR: 8.2), a median SUV_mean_ of 4.9 (IQR: 3.4), and a median SUV_min_ of 3.5 (IQR: 2.0). In meningiomas with partial transosseous involvement, the median volume was 5.9 cm³ (IQR: 10.3 cm³), with a median SUV_max_ of 15.8 (IQR: 14.7), a median SUV_mean_ of 7.9 (IQR: 4.7), and a median SUV_min_ of 4.6 (IQR: 0.9). In predominantly intraosseous meningiomas, the median volume was 1.3 cm³ (IQR: 7.2 cm³), with a median SUV_max_ of 9.3 (IQR: 8.8), a median SUV_mean_ of 5.3 (IQR: 4.0), and a median SUV_min_ of 3.3 (IQR: 2.1) (Fig. 3).

**Fig. 4:**
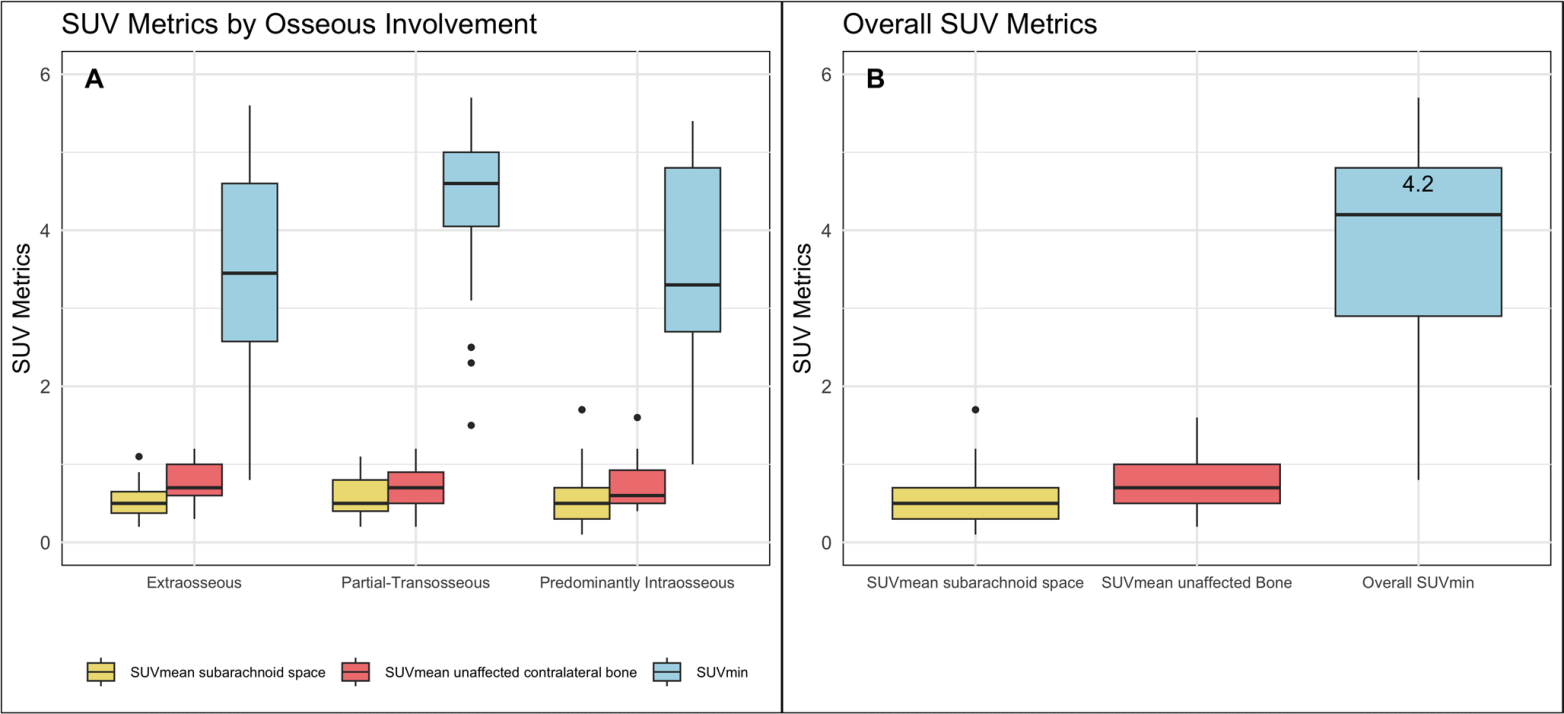
(**A**) Median optimal SUV threshold values (SUV_min_) for the delineation of meningioma lesions with different extensions, alongside median SUV_mean_ values for non-meningioma structures in the subarachnoid space and the unaffected bone of the contralateral side. This comparison did not reveal a significant difference in SUV_min_ between lesions with and without osseous involvement (Mann–Whitney U test, *p* = 0.1). (**B**) Overall SUV metrics

We compared the SUV_min_ values used for tumor delineation between meningiomas with and without osseous involvement. This comparison did not reveal a significant difference (Mann–Whitney U test, *p* = 0.1). The distribution of optimal SUV_min_ thresholds across lesion types is illustrated in Fig. 4. We also tested for differences in SUVmin values across anatomical locations using a Kruskal–Wallis test, which showed no statistically significant differences (H = 8.34, *p* = 0.138). Although suprasellar/parasellar lesions appeared to have numerically lower thresholds, the small sample size (*n* = 5) likely limited statistical power.

**Fig. 5:**
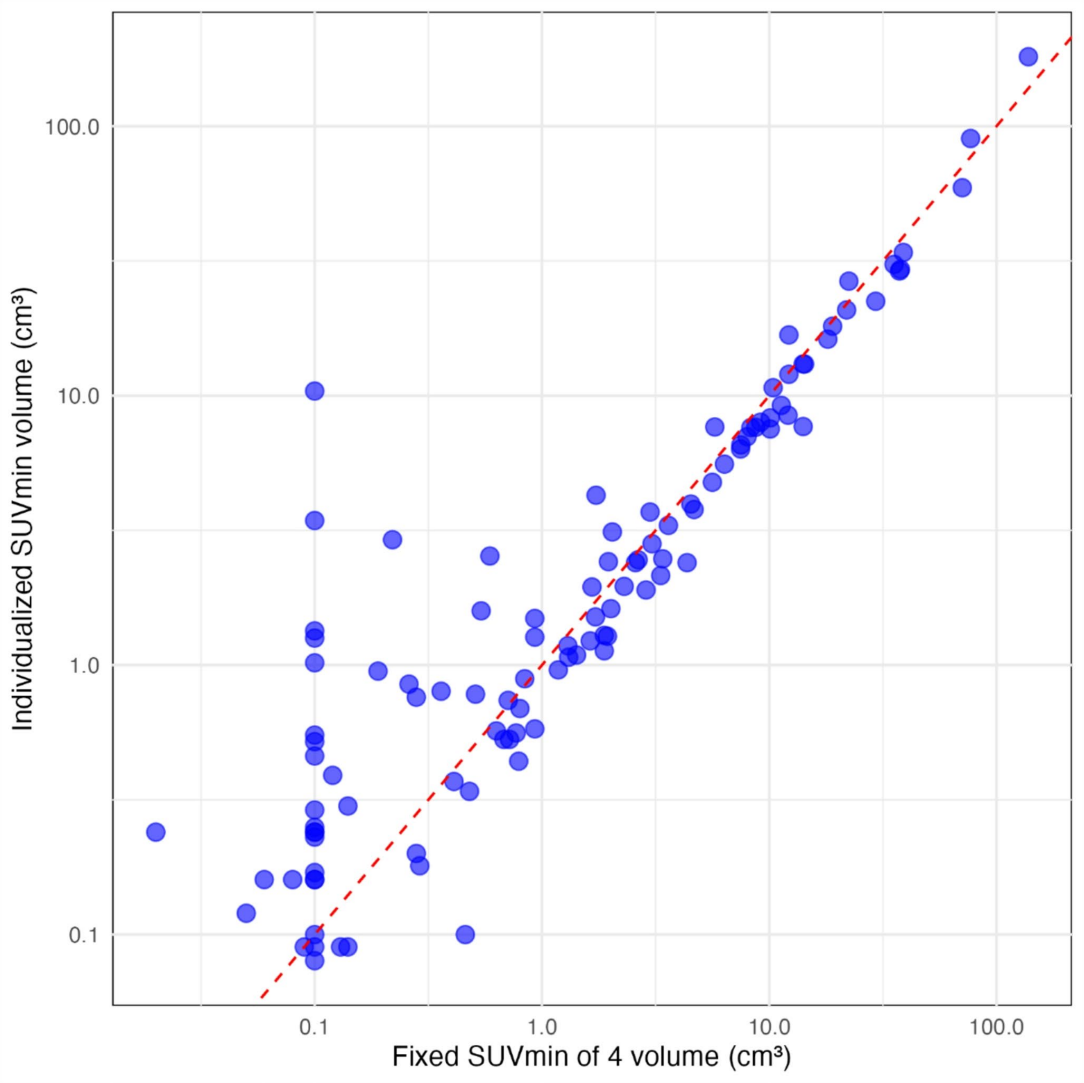
Scatter plot comparing lesion volumes for each individual lesion segmented using the individualized optimal SUV_min_ based on morphological comparison (y-axis) with volumes obtained using a fixed SUV cut-off of 4 (x-axis). Each blue dot represents an individual observation. The dashed red line represents equality, indicating identical values for both volume measurements. The intraclass correlation coefficient (ICC[A,1]) was 0.967 (95% CI: 0.952–0.977), indicating excellent agreement between the two methods. However, for very small lesions with volumes < 1 cm³, a divergence between the volumes becomes apparent

The individualized SUV volumes for each lesion, based on morphological comparison, showed excellent agreement with the volumes obtained using a fixed SUV threshold of 4, as assessed by the intraclass correlation coefficient (ICC[A,1] = 0.967; 95% CI: 0.952–0.977; *p* < 0.0001) (Fig. 5). However, a significant spread was observed for very small lesions with volumes < 1 cm³. Notably, 17 lesions, mostly very small, had an SUV_max_ below 4.2 and were therefore not captured by the fixed threshold.

### Characteristics of healthy organs

The median volume of the pituitary gland was 0.94 cm^3^ (IQR: 0.46 cm^3^), with a SUV_max_ of 21.1 (IQR: 13) and SUV_mean_ of 10 (IQR: 3.8). Both the median SUV_mean_ of the unaffected contralateral skull, at 0.7 (IQR: 0.5), and the SUV_mean_ of the unaffected subarachnoid space, at 0.5 (IQR: 0.4), were low, highlighting a strong lesion-to-background ratio (Fig. 4).

## Discussion

Establishing reliable SUV thresholds for [^18^F]SiTATE PET-based tumor segmentation is crucial for improving diagnostic accuracy and treatment planning in meningioma patients [18]. In this study, we initially performed semi-automatic segmentation of 109 lesions in PET imaging based on their correlation with CE-CT and CE-MRI to achieve the most precise delineation. Subsequently, we compared the volumes obtained using individualized SUV_min_ thresholds with those derived from a previously proposed fixed SUV threshold of 4. Interestingly, in our more heterogeneous cohort, the median optimal threshold (SUV_min_) for lesion delineation was 4.2, which is remarkably close to the proposed threshold of 4 [20]. Therefore, a strong correlation was observed between lesion volumes obtained using the individualized optimal thresholds based on morphological comparison and those segmented with the fixed SUV threshold of 4. Notably, segmentation was also successful in delineating meningiomas in complex postoperative cases, including transosseous and predominantly intraosseous disease. These cases are particularly challenging due to the difficulty of distinguishing intraosseous tumor extension, especially in the periorbital region, on CE-CT and CE-MRI [25]. However, no significant differences in SUV_min_ values were observed based on the extent of osseous involvement in our cohort, underscoring the general applicability of a fixed threshold (Table 2).

**Table 2:** PET/CT measurements of all meningiomas

Location	Total volume (cm^3^)	Osseous volume (cm^3^) *	Extraosseous volume (cm^3^)	Total SUV_max_	Total SUV_mean_	Total SUV_min_	Osseous SUV_max_ *	Osseous SUV_mean_ *
Parafalcine/Parasagittal (*n* = 25)	0.9 (3.4)	0.0	0.9 (3.4)	9.1 (9.3)	6.1 (3.1)	4.2 (1.5)	n/a	n/a
Convexity (*n* = 37)	1.1 (8.2)	0.5 (0.9)	0.7 (3.7)	7.4 (6.9)	5.1 (3.6)	3.5 (2.4)	6.2 (8.0)	4.6 (4.6)
Sphenoid wing (*n* = 31)	2.4 (7)	1.2 (3.1)	1.2 (4.3)	12.9 (11)	7.3 (4.2)	4.6 (1.5)	11.5 (11.4)	6.5 (6.5)
Cerebellopontine (*n* = 5)	1.6 (5.5)	0.1 (1.1)	1.6 (4.5)	21.4 (16.7)	10.1 (7.9)	4.7 (1.8)	8.8 (14.7)	6.2 (4.9)
Suprasellar/Parasellar (*n* = 5)	2.6 (3.2)	0.1 (0.4)	1.3 (2.8)	7.1 (16.9)	3.9 (6.9)	2.6 (2.0)	5.3 (13.7)	3.4 (7.3)
Skull (intraosseous) (*n* = 6)	1.2 (5.1)	0.4 (1.9)	0.8 (3.2)	13.8 (7.6)	5.9 (1.7)	3.2 (0.7)	11.2 (5.9)	5.3 (0.4)

Values are presented as median (IQR). The *p*-value was calculated using Kruskal-Wallis test. Significant differences (*p* < 0.05) are indicated with an asterisk*. SUV_min_ indicated the median optimal threshold used for meningioma delineation in PET/CT. n/a = not applicable; no osseous component present in Parafalcine/Parasagittal meningiomas

Nevertheless, it is important to note that using a fixed threshold can lead to deviations from the true lesion volume. In our cohort, 17 lesions with an SUV_max_ below 4 were not captured by the fixed threshold. These were primarily small lesions (< 2 ml), supporting the assumption that they are of limited clinical significance. This is in line with the limited but suggestive literature indicating that smaller meningiomas are less likely to be symptomatic or require treatment, e.g. in a prior study a threshold tumor volume of 21.1 ml was associated with the likelihood of symptom development [26]. However, one lesion in our cohort warrants particular attention: a 10.3 ml meningioma that was not detected using the fixed threshold due to its unusually low uptake (SUV_max_ 3.9). Interestingly, histopathology confirmed this lesion to be a transitional meningioma, underscoring that even histologically intermediate subtypes may exhibit reduced tracer avidity. While rare, such cases highlight the importance of visual inspection of not only the PET component and the potential need for individualized adjustments in threshold-based segmentation, particularly when dealing with atypical tumor biology. Furthermore, in specific scenarios, such as planning radiotherapy margins or longitudinal volumetric monitoring, even small lesions could be relevant. It should be noted here that small lesions generally limit the effectiveness of PET/CT as a method for optimal segmentation, as they are susceptible to subvolume and spillover effects [27]. As such, in clinical practice, visual assessment and flexible manual adjustment of thresholds remain important for comprehensive evaluation, particularly in borderline or ambiguous cases. This highlights the need for threshold-guided segmentation to be integrated into a broader interpretative framework rather than used as a strict stand-alone metric.

It is also noteworthy that the fixed threshold of 4 did not lead to a significant overestimation of lesion volume in our cohort (Fig. 5). This highlights the high diagnostic accuracy of [^18^F]SiTATE PET/CT in differentiating meningiomas from surrounding tissue, even in recurrent tumors after previous therapy and in various anatomical locations (Fig. 4).

Our findings can be contextualized by comparing them to previous studies using [⁶⁸Ga]Ga-DOTATATE and [^68^Ga]Ga-DOTATOC PET, which is currently more widely studied in meningioma imaging. Several studies have proposed SUV-based criteria using [⁶⁸Ga]-labeled tracers, including absolute SUV thresholds and SUV ratios to different reference region, such as an SUV of 2.3 for differentiating meningioma from non-neoplastic tissue [12, 28]. However, these thresholds were derived under different imaging conditions and using a different tracer with distinct physical properties, including a shorter half-life and lower positron yield compared to [¹⁸F]SiTATE. These differences affect image resolution and quantification accuracy, which may partly explain the higher SUV threshold observed in our study. Moreover, unlike [⁶⁸Ga]-based studies that mostly included untreated, well-delineated extraosseous meningiomas, our cohort encompassed a broader spectrum of lesion types and clinical scenarios.

Since SUV accuracy can be affected by factors like noise, image resolution, and ROI definition, fixed thresholds should not be regarded as rigid values but rather as a practical tool to simplify clinical workflows. They provide an initial approximation for semi-automatic segmentation in therapy planning or follow-up, which can then be adjusted on a case-by-case basis to align with true tumor boundaries. This approach is particularly valuable given the inherent challenges of accurately delineating meningiomas due to the physical limitations of PET imaging, such as physiologically high [^18^F]SiTATE uptake in adjacent structures like the pituitary gland or the presence of rare SSTR2-negative meningiomas [29, 30]. Despite these complexities, having an approximation method to accurately determine meningioma extent in routine clinical practice remains of significant value [18].

Although our results support the utility of a fixed SUV threshold in the context of meningiomas imaged with [¹⁸F]SiTATE these findings cannot be directly extrapolated to other tumor entities or SSTR-targeted tracers. Tracer kinetics, biodistribution, and receptor expression profiles vary substantially between tracers and tumor types. Therefore, threshold values are likely to be context- and tracer-specific.

There are limitations to this analysis: First, it is a single-center study conducted with a standardized protocol on two PET/CT devices from one vendor, which inherently limits the generalizability of the findings. Although previous studies have suggested that differences in PET scanners and reconstruction algorithms affect the reproducibility of SUV_max_, reconstruction methods can also significantly influence tumor-to-background ratios and, importantly, volumetric tumor measurements [31]. Therefore, our threshold findings must be interpreted cautiously when applied to other scanners and reconstruction protocols. Future multicenter studies specifically addressing these factors will be essential to validate and refine our findings. Second, this study is purely imaging-based. More studies involving larger cohorts and correlating imaging findings with histology, preferably using stereotactic sampling, are warranted to further validate and refine the proposed thresholds. Such research will help solidify the role of [^18^F]SiTATE PET/CT in the clinical management of meningiomas.

## Conclusion

While prior studies on SUV thresholds for [^18^F]SiTATE PET-based tumor segmentation focused on untreated extraosseous meningiomas with narrow inclusion criteria, our study broadens the perspective by including extra-, trans-, and intraosseous lesions, as well as cases with prior surgical intervention. This expanded scope supports the potential utility of fixed SUV thresholds across a more diverse range of clinical scenarios. The recently proposed SUV threshold of 4 demonstrated promising results for precise tumor delineation, even in challenging anatomical settings and a heterogeneous patient cohort, validating its suitability for integration into clinical practice. However, limitations were evident in very small meningiomas underscoring the need for careful consideration of threshold-based segmentation in such cases. Overall, this study highlights the utility of fixed SUV thresholds as practical tools in clinical workflows and supports further validation in larger, multicenter cohorts to refine their generalizability and applicability.

## Data Availability

The datasets generated during and/or analysed during the current study are available from the corresponding author on reasonable request.
